# Perceived roles, benefits and barriers of virtual global health partnership initiatives: a cross-sectional exploratory study

**DOI:** 10.1186/s41256-022-00244-4

**Published:** 2022-04-28

**Authors:** Lisa Umphrey, George Paasi, William Windsor, Grace Abongo, Jessica Evert, Heather Haq, Elizabeth M. Keating, Suet Kam Lam, Megan S. McHenry, Carolyne Ndila, Charles Nwobu, Amy Rule, Reena P. Tam, Daniel Olson, Peter Olupot-Olupot

**Affiliations:** 1 Department of Pediatrics, University of Colorado School of Medicine, 13123 E 16th Ave, B302, Aurora, CO 80045 USA; 2 Center for Global Health, Colorado School of Public Health, 13199 E Montview Blvd, Ste 310, A090, Aurora, CO 80045 USA; 3 Mbale Clinical Research Institute, Plot 29, 33 Pallisa, Mbale, Uganda; 4 Child Family Health International, 11135 San Pablo Ave #929, El Cerrito, CA 94530 USA; 5 Department of Pediatrics, Baylor College of Medicine, 1 Baylor Plaza, Houston, TX 77030 USA; 6 Division of Pediatric Emergency Medicine, University of Utah School of Medicine, 30 N 1900 E, Salt Lake City, UT 84132 USA; 7 Cleveland Clinic Lerner College of Medicine, Case Western Reserve University School of Medicine, EC-10 Cleveland Clinic, 9501 Euclid Ave, Cleveland, OH 44195 USA; 8 Department of Pediatrics, Indiana University School of Medicine, 340 W 10th St, Indianapolis, IN 46202 USA; 9 Child Family Health International, Accra, Ghana; 10 Cincinnati Children’s Hospital Medical Center, University of Cincinnati College of Medicine, 3333 Burnet Ave, Cincinnati, OH 45229 USA; 11 Department of Pediatrics, University of Utah School of Medicine, 30 N 1900 E, Salt Lake City, UT 84132 USA; 12 Busitema University, P.O. Box 1460, Mbale, Uganda

**Keywords:** Global health, Partnership, Virtual, Pandemic

## Abstract

**Background:**

Virtual global health partnership initiatives (VGHPIs) evolved rapidly during the COVID-19 pandemic to ensure partnership continuity. However the current landscape for VGHPI use and preference is unknown. This study aimed to increase understanding of GH partners’ perspectives on VGHPIs.

**Methods:**

From 15 October to 30 November 2020, An online, international survey was conducted using snowball sampling to document pandemic-related changes in partnership activities, preferences for VGHPIs, and perceived acceptability and barriers. The survey underwent iterative development within a diverse author group, representing academic and clinical institutions, and the non-profit sector. Participants from their professional global health networks were invited, including focal points for global health partnerships while excluding trainees and respondents from the European Economic Area. Analysis stratified responses by country income classification and partnership type. Authors used descriptive statistics to characterize responses, defining statistical significance as α = 0.05.

**Results:**

A total of 128 respondents described 219 partnerships. 152/219 (69%) partnerships were transnational, 157/219 (72%) were of > 5 years duration, and 127/219 (60%) included bidirectional site visits. High-income country (HIC) partners sent significantly more learners to low- to middle-income country (LMIC) partner sites (*p* < 0.01). Participants commented on pandemic-related disruptions affecting 217/219 (99%) partnerships; 195/217 (90%) were disruption to activities; 122/217 (56%) to communication; 73/217 (34%) to access to professional support; and 72/217 (33%) to funding. Respondents indicated that VGHPIs would be important to 206/219 (94%) of their partnerships moving forward. There were overall differences in resource availability, technological capacity, and VGHPI preferences between LMIC and HIC respondents, with a statistically significant difference in VGHPI acceptability (*p* < 0.001). There was no significant difference between groups regarding VGHPIs’ perceived barriers.

**Conclusions:**

The pandemic disrupted essential partnership elements, compounding differences between LMIC and HIC partners in their resources and preferences for partnership activities. VGHPIs have the potential to bridge new and existing gaps and maximize gains, bi-directionality, and equity in partnerships during and after COVID-19.

**Supplementary Information:**

The online version contains supplementary material available at 10.1186/s41256-022-00244-4.

## Introduction

Global Health (GH) is a rapidly growing field focused on advancing international and interdisciplinary healthcare [1–12]. A core tenet of GH is addressing health inequities [13], and one central strategy of this approach is the implementation of Global Health Partnerships (GHPs), defined as a collaborative effort towards training, research, and capacity building [1, 14].

The heterogeneity across diverse settings, individual preferences, and organizational interests are factors that determine the nature, duration, and future of GHPs. Moreover, the stages of development and maturity of GHPs are dependent on the complexity of and investment in activities undertaken locally and internationally [1–12]. The focus areas of GHPs range from electives for healthcare trainees; access to resources; capacity building; technical support; professional mentorship; and collaborative research, public health, or education initiatives [1, 5–7, 14–19]. Many GHPs rely heavily on unidirectional travel, with faculty and trainees from high-income countries (HIC) disproportionately visiting host sites in low- and middle-income countries (LMIC) [1, 6, 7, 14–16, 20–24]. Although bidirectional exchanges of information, resources, and personnel are considered ideal [1, 2, 6, 7, 14, 15, 20, 22, 25], implementing these and measuring their impact are often prohibitively expensive and complicated [6].

The COVID-19 pandemic disrupted in-person GHP activities while consequently worsening the existing inherent inequities in GH, especially in LMIC [26, 27]. Since the start of the COVID-19 pandemic, traditional GHP operations have been affected through unprecedented travel restrictions, limitations of in-person interactions, and new standards for personal protection [16, 26, 28–30]. Challenges to traditional approaches to GHPs, including communication, bidirectional exchange of staff and learners, and in-person site visits, have continued throughout the pandemic. Beyond that, hindrances due to financial constraints, visa entry tied to COVID-19 risk or vaccination status, and GHP programing may continue to limit in-person GHP activities [16, 29]. Concerted efforts to ensure GHP continuity are therefore paramount. Consequently, there is an urgent need for GHPs to re-examine how they will operate during the pandemic and beyond [31].

To date, there has been little research dedicated to virtual approaches to GHPs, and there are few data on ways to sustain or improve GHPs partnerships during disruptive global challenges, such as a pandemic. Guidance exists on the use of virtual education for GH preparation, simulation, and educational initiatives [7, 15, 32–35], most commonly focusing on an individual activity, however less clear is how to maintain activities during the pandemic. Further, learner competencies for GH education exist [1, 32–35], but recommendations on how to reinforce competencies virtually or by distance while prioritizing the needs of partners in LMIC [16, 28, 36] are lacking. Finally, few papers compare different virtual engagement strategies or document preferences for or enablers and barriers to those strategies, particularly from the LMIC partner perspective. Virtual GHP initiatives (VGHPIs), which we define as collaborative GHP activities conducted entirely online, may inform approaches for re-organization and re-prioritization of GH activities.

VGHPIs will not only enable continuity of GHP activities during COVID-19 pandemic but may also improve upon standard GHP practices going forward; as such, baseline data will be essential in guiding future discussions and establishing future best practice recommendations. Therefore, the aim of this study was to generate preliminary descriptive data about VGHPIs that can direct future study and inform ongoing discussions about approaches for re-organization and re-prioritization of GH activities.

## Methods

### Setting and study design

We conducted a cross-sectional, online and international survey. The survey goal was to characterize GHPs and their practices, assess changes in activities during the COVID-19 pandemic, document perceived acceptability of and barriers to VGHPIs, and enrich our understanding of alternative strategies to maintain GHPs.

Our exploratory survey targeted a broad audience to help identify key topic areas. Eligible participants were faculty or staff who self-identified as key members of GHPs at an organization. Ineligible participants were people not directly involved in a GHP at an organization and trainees. Because of data protection restrictions impacting European Economic Area (EEA) countries and a resultant longer ethical review period, we excluded EEA respondents. If respondents served as a focal point for more than one GHP, a common practice of multinational organizations or those in academia with ties to external organizations, they could respond on behalf of up to three unique partnerships within the same survey.

We implemented snowball sampling to access a wide range of participants in the authors’ professional networks. We invited survey participation by email correspondence from each author to known focal points for GHPs (i.e., program directors, GH pathway directors, organization presidents/directors, student organization focal points, and/or clinical directors/supervisors). We also contacted listserv operators (such as one for GH residency track and fellowship directors), and GH-related social media pages (such as the twitter account for an upcoming GH conference). Each invitation included a request to circulate the survey link to people both within and external to a participant’s organization.

### Survey development

Our team, which included members of diverse academic, non-profit, and clinical organizations, developed survey questions based on literature review and an iterative process until reaching consensus among the author group. We structured Likert scale questions to elicit baseline data on respondents’ demographics, GHP characteristics, impressions of acceptability and barriers of VGHPIs, and preferences for types of VGHPIs. We included both closed-ended and free-text questions, and we allowed participants to comment on up to three GHPs. The survey and all study materials were translated from English into French and Spanish.

Expanding on previous definitions of GH [2–4, 9–12, 18, 26], for the survey, we defined a GHP as “*any trans- or multinational and/or domestic collaborative health partnership that bridges geographical distance and/or resource levels to promote the health and wellbeing of people anywhere in the world*.” Partnerships could include headquarter or satellite sites of a single entity or represent individual organizations linked by the coalition. The collaboration could include clinical, public health, research, community, educational, and/or development work, but each were required to share a mutual focus to advance health within the capacities of their respective organizations.

### Data collection and processing

The survey was open from 15 October to 30 November 2020. After the initial invitation to participate, we sent reminder emails twice during the data collection period to encourage participation. We gathered data via an online survey created and stored via the standard encrypted online cloud platform for data collection and storage, REDCap (Vanderbilt University, http://project-redcap.org) [37]. Participants received the REDCap survey link, a standard study information leaflet sheet, and study contact details. The link directed participants to a survey introduction and explanation page, and respondents who consented to participate continued to the survey. We de-identified all survey responses.

A total of 134 respondents from 34 countries completed the survey. Five surveys were excluded due to respondents being in the EEA and one response was excluded due to missing location. Among respondents, 115/128 (90%) completed the survey in full, while 13/128 (10%) responses were partial.

### Data analysis

We stratified responses by type of GHP (domestic, transnational, or both/blended) and by income status (HIC vs. LMIC) [38] of the participant’s country of professional work. Using SAS software, version 9.4 (SAS Institute Inc., Cary, NC, USA), we summarized descriptive data using frequencies for dichotomous and categorical variables and measures of central tendency for continuous variables. We analyzed bivariate relationships between GHPs using chi-square tests for categorical variables. We determined statistical significance with an alpha of 0.05. We did not implement qualitative analysis of free-text responses as we did not receive adequate qualitative data in these portions of the survey.

### Ethical considerations

We obtained institutional review board approval from the Colorado Multiple Institution Review Board (University of Colorado, Aurora, CO, USA; #20-2099) and the Mbale Regional Referral Hospital Research Ethics Committee (Mbale, Uganda; #MRRHREC-OUT-011/2020).

## Results

### Respondent characteristics

Table 1 shows respondent characteristics. Geographical regions represented included North America (65/128, 51%; USA), Africa (39/128, 30%; Botswana, Democratic Republic of Congo, Ethiopia, Gambia, Ghana, Kenya, Lesotho, Malawi, Morocco, Nigeria, Rwanda, Sierra Leone, South Africa, Tanzania, Uganda, Zambia), South/Latin America (12/128, 9%; Argentina, Colombia, Dominican Republic, Ecuador, Guatemala, Haiti, Mexico, Nicaragua), Asia Pacific (7/128, 6%; India, Indonesia, Japan, Philippines), and the Middle East (5/128, 4%; Lebanon, Pakistan, Sudan). Among respondents, 62/128 (48%) were from LMIC while 66/128 (52%) were from HIC, and 79/128 (62%) were from health centers/hospitals. Respondents reported that 55/128 (43%) of their organizations focused on district/county/region level work. Respondents were permitted to indicate multiple roles or specialties within the same institution. They represented a wide range of institutional roles, with 68/128 (53%) working as professor/educators; among clinician respondents, 26/128 (20%) worked in pediatrics, the most common specialty represented.

**Table 1:** Characteristics of eligible respondents to virtual global health partnership initiative survey

Characteristic	Variable	N (%)
Respondent region	North America	65 (51)
	Africa	39 (30)
	South America	12 (9)
	Asia and Pacific	7 (6)
	Middle East	5 (4)
Respondent’s institution type	Healthcare center or hospital	79 (62)
	Health profession school	58 (45)
	Research organization	26 (20)
	Non-government organization	23 (18)
	Public health or community service organization	12 (9)
	Governmental agency	10 (8)
	Other	7 (5)
Geographical reach of respondent’s organization	District/county/region	55 (43)
	Village/town/city	40 (31)
	National	38 (30)
	Multi-continental	35 (27)
	Multi-regional/country	21 (16)
Respondent role	Professor or educator	68 (53)
	Clinical staff	57 (45)
	Researcher	38 (30)
	Director/president of organization	29 (23)
	Administrator	25 (20)
	Other	11 (9)
Respondent clinical discipline, if applicable	Pediatrics	26 (20)
	Emergency medical services	11 (9)
	Internal medicine	6 (5)
	General medicine	5 (4)
	Surgery	3 (2)
	Obstetrics/gynecology	2 (2)
	Other	8 (6)

### Global health partnership characteristics

While 51/128 (40%) of respondents completed the survey for one GHP, 77/128 (60%) respondents served as a focal point for multiple GHPs. Subsequently our dataset represents 219 total GHPs. Table 2, Part A describes characteristics of these GHPs.

**Table 2:** Global health partnerships—characterizations and virtual activities

		Partnership type			
		Domestic partnerships	Transnational partnerships	Blended partnerships	*p* value
		N = 30 (%)	N = 152 (%)	N = 37 (%)	
Part A: Characteristics of global health partnerships					
Nature of partnerships	Partnerships within country	25 (83%)	8 (5%)	32 (86%)	< 0.001*
	Partnerships with LMIC-based organizations	4 (13%)	115 (76%)	31 (84%)	< 0.001*
	Partnerships with HIC-based organizations	4 (13%)	29 (19%)	28 (76%)	< 0.001*
Length of partnerships	< 5 years	11 (37%)	42 (28%)	4 (24%)	0.009*
	5–10 years	14 (47%)	50 (33%)	6 (16%)	
	> 10 years	5 (17%)	60 (39%)	22 (59%)	
Partnership activity types	Research	19 (63%)	101 (66%)	33 (89%)	0.018*
	Education	19 (63%)	120 (79%)	32 (86%)	0.07
	Clinical	12 (40%)	94 (62%)	20 (54%)	0.08
	Community Development	11 (37%)	31 (20%)	17 (46%)	0.003*
Physical interaction of partnership staff between sites?	Yes (%)	23 (77%)	137 (90%)	32 (86%)	0.12
Organization country income status	Low/middle income	26 (87%)	38 (25%)	34 (92%)	< 0.001*
	High income	4 (13%)	114 (75%)	3 (8%)	
Part B: Virtual activities within global health partnerships					
Did your organization offer virtual partnership activities prior to December 2019?	Yes	12 (40%)	48 (32%)	24 (67%)	< 0.001*
	No	13 (43%)	85 (56%)	7 (19%)	
	Planned but not implemented	4 (13%)	17 (11%)	5 (14%)	
What kinds of virtual partnership activities were you previously engaged in?	Access to online educational materials	5 (17%)	29 (19%)	15 (40%)	< 0.001*
	Virtual face to face educational training	6 (20%)	31 (20%)	21 (57%)	< 0.001*
	Virtual face to face clinical care	1 (3%)	8 (5%)	12 (32%)	< 0.001*
	Virtual peer to peer support	4 (13%)	20 (13%)	10 (27%)	0.11
	Research Collaborations	5 (17%)	21 (14%)	9 (24%)	0.29
How often did these activities occur?	Daily	0 (0%)	3 (6%)	2 (9%)	0.25
	Weekly	5 (42%)	16 (34%)	10 (43%)	
	Monthly	3 (25%)	15 (32%)	8 (35%)	
	Quarterly	4 (33%)	5 (11%)	1 (4%)	
	Yearly	0 (0%)	1 (2%)	2 (9%)	
	No regular frequency	0 (0%)	6 (13%)	0 (0%)	
Would new or ongoing virtual health partnerships be important to your organization?	Yes (%)	27 (90%)	145 (95%)	34 (100%)	0.16
How?	Enable continuity of activities between partners	18 (82%)	102 (71%)	34 (100%)	0.002*
	Guide planning for virtual collaborative initiatives	0 (0%)	15 (10%)	0 (0%)	
	Allow for safer partnerships	1 (5%)	20 (14%)	0 (0%)	
	Allows for career advancement	3 (14%)	6 (4%)	0 (0%)	

*Statistically significant distribution at *p* value < 0.05

Of the 219 GHPs represented, 152/219 (69%) were transnational (partnerships between different countries); 30/219 (14%) were domestic (partnerships within one country); and 17% 37/219 (17%) were blended (partnerships with both domestic and transnational activities). Of domestic GHPs, 26/30 (87%) described partnerships in which both members were based in a LMIC, while 4/30 (13%) described partnerships in which both members were based within a HIC. The majority of GHPs described were > 5 years old, with the longest partnerships between blended partnership types. The highest proportion of GHP activities reported were research and education. Domestic and blended GHPs were primarily based in LMIC, while transnational GHPs were primarily based in HIC.

Of all GHPs described, 127/219 (60%) included bidirectional site visits while 79/219 (40%) included unidirectional site visits. Among GHPs that included bidirectional site visits, we found that HIC partners sent significantly more learners to LMIC partner sites than LMIC partners sent to HIC sites (36% vs. 20% respectively, *p* < 0.001). Among GHPs that included unidirectional site visits, significantly more LMIC sites only hosted visitors when compared to HIC partners (16% vs 4% respectively, *p* < 0.001).

### Pandemic-related disruptions to global health partnership

Participants commented on pandemic-related disruptions to 217/219 (99%) GHPs, 97 (45%) from the LMIC partner and 120 (55%) from the HIC partner. Among GHPs described, 195/217 (90%) reported a significant disruption in activities; 122/217 (56%) in communication; 73/217 (34%) in access to professional support and resources; and 72/217 (33%) in funding. Overall, more respondents from LMIC versus HIC reported disruption to partnership activities due to the pandemic (*p* = 0.005). Of partners in LMIC, 53/97 (55%) reported disruptions in funding, compared to only 19/120 (16%) partners in HIC (*p* < 0.001). LMIC partners reported significantly more disruption in access to professional support and resources than GH partners in HIC (54% vs 18% respectively, *p* < 0.001). Disruption in communication with partners was similar for both LMIC and HIC respondents (57% vs 55% respectively, *p* = 0.75).

### VGHPI interest and preference

Table 2, Part B presents virtual activities within GHPs and future interest in VGHPIs. Before the onset of the pandemic, 84/219 (38%) GHPs engaged in VGHPIs, most commonly occurring weekly. Respondents indicated that in the future, VGHPIs would be important for 206/219 (94%) of their GHPs; the ways in which VGHPIs could be important, however, differed by partnership type.

**Fig. 1:**
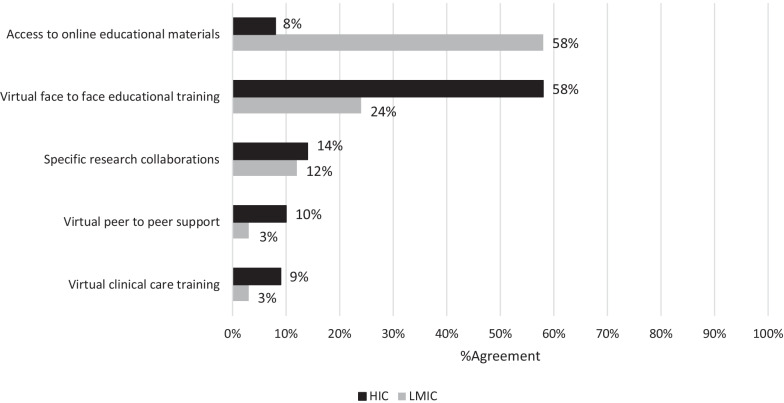
Most highly preferred virtual global Health partnership initiatives (VGHPIs) during the COVID-19 pandemic

Figure 1 shows the most preferred VGHPIs during the COVID-19 pandemic among GHP sites stratified by country-level income status. We found a significant difference between LMIC and HIC respondents in the distribution of all preferred VGHPIs (*p* < 0.001).

### VGHPI technological capacity

**Fig. 2:**
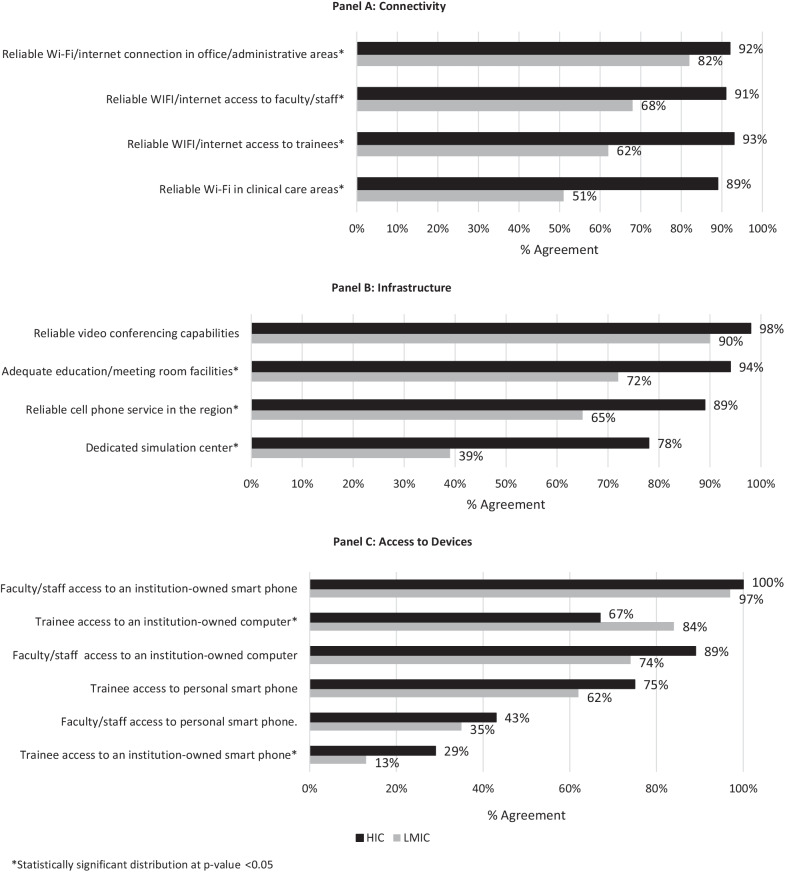
Technological capacity for virtual global health partnership initiatives (VGHPIs) among respondents

Figure 2 shows the status of technological capacity in respondents’ home organization, stratified by connectivity, available infrastructure, and device accessibility. When comparing HIC and LMIC respondents, there were statistically significant differences between accessibility to a variety of resources.

### VGHPI perceived barriers and attitudes

Table 3 Panel A shows perceived barriers and attitudes about VHGPIs. There was no significant difference between LMIC and HIC respondents on their perceptions of barriers to VGHPIs. Regarding acceptability of VHGPIs (Table 3 Panel B), LMIC and HIC respondents differed significantly in their perceptions that they needed additional education to succeed with VGHPIs (48% and 14% respectively; *p* < 0.001); for preference of in-person activities versus VGHPIs (51% and 74%, respectively; *p* = 0.02); for requiring technical support to adequately participate in VGHPIs (53% and 22% respectively; *p* < 0.001); and for ease of participating in VGHPIs (66% and 39% respectively; *p* = 0.006). LMIC respondents were more likely to agree that virtual applications and technology were complicated compared to HIC respondents (39% and 19%, respectively; *p* = 0.04). Figures 3a and 3b (Additional file 1) further summarize the perceived barriers and acceptability of VGHPIs. There were no significant differences between respondents when stratified by the three most common types of participant home institution.

**Table 3:** Perceived barriers and acceptability of virtual global health partnership initiatives (VGHPIs) among respondents

	LMIC	HIC	*p* value
	N = 61 (%)	N = 66 (%)	
Panel A: Perceived barriers of VGHPIs			
Lack of institution accreditation/ acknowledgement	36 (60%)	35 (55%)	0.66
Lack of space/facilities	37 (62%)	32 (49%)	0.19
Lack of specialty accreditation/ acknowledgement	35 (58%)	30 (47%)	0.42
Lack of technological capacity	41 (68%)	36 (55%)	0.16
Physical participation/ interaction requirement	41 (68%)	39 (61%)	0.66
Time needed to train staff	43 (72%)	56 (86%)	0.06
Cost of training	45 (75%)	42 (65%)	0.44
Lack of institutional support	45 (75%)	48 (75%)	0.58
Lack of mentors/local champions	47 (78%)	51 (78%)	0.57
Lack of onsite technical support	48 (80%)	45 (70%)	0.27
Lack of equipment for virtual education	49 (82%)	46 (71%)	0.19
Lack of formal training curriculum	49 (82%)	49 (78%)	0.51
Panel B: Acceptability/Usability of VGHPIs			
Starting VGHPIs			
I must learn many things to succeed with VGHPIs	28 (48%)	9 (14%)	< 0.001*
I prefer in-person activities over virtual partnership activities	30 (51%)	48 (74%)	0.02*
I would feel confident in participating in VGHPIs	48 (81%)	59 (91%)	0.28
I think my organization is interested in collaborating to expand VGHPIs	49 (83%)	49 (75%)	0.55
I want to implement virtual partnerships	52 (88%)	56 (88%)	0.62
Participation in VGHPIs			
I need technical support staff to adequately participate	31 (53%)	14 (22%)	< 0.001*
I think VGHPI rollout would go smoothly	37 (63%)	30 (46%)	0.14
I anticipate continuing VGHPIs in the future	54 (92%)	63 (97%)	0.19
Utilization of VGHPIs			
I think VGHPIs would be awkward/uncomfortable	12 (20%)	7 (11%)	0.22
I think VGHPIs will be inconsistent at my organization	19 (32%)	23 (35%)	0.60
I think virtual applications and technology are complicated	23 (39%)	12 (19%)	0.04*
I think participation in VGHPIs will be easy	39 (66%)	25 (39%)	0.01*
I think VGHPIs can be implemented quickly	40 (68%)	34 (52%)	0.19
I think VGHPIs would complement existing activities	56 (95%)	61 (94%)	0.35

*VGHPI* virtual global health partnership initiative, *LMIC* low/middle income country, *HIC* high income country

*Statistically significant distribution at *p* value < 0.05

## Discussion

To our knowledge, our study is the first to explore the role of VGHPIs and their perceived benefits and barriers for the resilience of GHPs during the COVID-19 pandemic. Further, our survey is the first to query current LMIC and HIC members of GHPs to document initial preferences for and interest in VGHPIs, an important first step in a series of future studies to strengthen global partnerships. Although our baseline data are descriptive only, our data contribute critical information to advance previous discussions about supporting partners in LMIC during global challenges, such as epidemics or crises [10, 43] and lay the groundwork to discuss solutions to challenges posed by shifting to virtual engagement. Additionally our findings add a real-world perspective to recent discussions about shifting GHP activities virtually [16, 28, 36, 39], addressing LMIC partner needs thoughtfully [30], addressing virtual education needs within certain specialties [19, 26, 29], and proposing virtual programming relevant only in HIC [40, 41].

Despite our small sample size of 128 people, respondents represented multiple types of institutions active within 34 countries, and participants shared details for up to three unique GHPs for which they were a focal point. This allowed for an expanded dataset within a relatively limited participant group. The GHPs included in this study are like those described previously [1, 6, 7, 14–16, 20–25], and our data offer insights into VGHPI considerations for similar GHPs. A few GHPs in our dataset represented domestic LMIC/LMIC or HIC/HIC partnerships, “global local” pairings whose unique needs should be considered during implementation of VGHPIs [1, 2, 8]. Most partnerships were bidirectional, but the reported exchanges, whether bidirectional or unidirectional, were mainly from the HIC to the LMIC partner. Previously described successful bidirectional and collaborative initiatives during crises [15, 42] may be just as (or more) easily done virtually and favor the needs of the LMIC partner. Such transferable activities include opening access to educational resources; connecting subspecialists from HIC to LMIC sites; assisting LMIC faculty with grant writing and budget preparation; assisting LMIC trainees with residency application or entry examination preparation; offloading administrative tasks from the LMIC partner to HIC partner; and advocating for funds to improve LMIC partners’ administrative, office and technological capacity. VGHPIs provide an opportunity for complementing and coordinating efforts in GHPs, a tenet of ethical GH practices [43], more efficiently than ever before.

Not surprisingly, a minority of GHPs were engaged in VGHPIs before the pandemic, often with a weekly frequency. Thus, future studies looking at ideal activity frequency and ongoing activity preferences will be helpful in providing best practice recommendations. Interestingly, the respondents reported that VGHPIs would be important to the vast majority (206/219, 94%) of their GHPs moving forward. The significant differences in opinion about how VGHPIs would be important for domestic, transnational, and blended GHPs will be key discussion points for partnerships looking to incorporate or expand VGHPIs. Although we only inquired about four ways in which VGHPIs may be important (enabling continuity of activities vs usefulness in guiding planning vs increasing safety of partnerships vs allowing for career advancement), our findings suggest that partners’ priorities and needs may not be aligned, and shared priorities should not be assumed. Likewise, data showing the significant differences between HIC and LMIC partners regarding preferred types of VGHPIs, most specifically in terms of access to online materials (preferred by LMIC more than HIC respondents) and valuing virtual face-to-face trainings (preferred by HIC more than LMIC respondents), are telling. Because HIC and LMIC partners seem to value different components of VGHPIs, these notable differences should prompt ongoing and intentional discussions to ensure all parties are mutually benefitting from VGHPI implementation and roll out.

Our data show ongoing discrepancies between resource access and allocation that worsened since the pandemic began. For example, most respondents reported pandemic-related disruptions in communication at their GHP sites, but disruptions in funding, partnership activities, and access to professional support and resources were significantly more disruptive for LMIC compared to HIC respondents. This must be taken into consideration for future emergency responses and in how VGHPIs are structured and planned from baseline.

Regarding barriers to VGHPIs, although there were no significant differences between LMIC and HIC respondents on perceived barriers to VGHPIs, the agreement between respondents could counter assumptions that members of GHPs may make about each other. The time needed for training and the lack of training curriculum were among the most frequently reported barriers by GHP sites. This suggests underlying healthcare system challenges in enabling continuous professional education within the GHPs. Regarding technological capacity, our findings agree with previous studies [1, 8, 14, 36, 44–46] that suggest a lack of internet connectivity is a severe concern for GHPs, with important implications for VGHPIs. We found that LMIC partners reported less access to wireless internet, less trainee access to organization-owned hardware, poorer cellular phone service, and less access to physical spaces like meeting and simulation facilities. However, both HIC and LMIC respondents had reliable access to personal smartphones, to organization-owned technology, and video-conferencing services. Considering the technological capacity within GHPs and possibly investing into communication infrastructure will be critical to ensure successful virtual engagement. Funding for in-person activities could be shifted towards resources that improve internet connectivity at LMIC partner-sites to address this challenge.

Between the LMIC and HIC partners, there were several key differences in opinion about VGHPI acceptability. Significantly more respondents in LMIC compared to HIC reported they would need to learn many things to succeed with VGHPIs and require technical support to fully participate in VGHPIs. This need for education and support must be considered moving forward to ensure the needs of LMIC partners are adequately heard and met. Interestingly, more LMIC respondents reported that participating in VGHPIs would be easy, but that virtual applications and technology are complicated. This perhaps reflects the difference between using technology (something many LMIC partners are accustomed to as the hosting partner in a GHP) versus reliable access to technology (reflecting challenges in technological capacity), which indicates that reliable access must be accompanied by reliable training. This could be further examined in future studies.

VGHPIs thrived since the start of the pandemic, but their success depended on the environment and partnership strengths where they occurred. We recommend that each unique GHP now engage in thoughtful, frank conversations about VGHPIs—addressing previous partnership issues while identifying GHP strengths—to decide how to bring virtual engagement equitably into their activities. Discussions should include what administrative, logistic, or technologic support each partner may need moving forward, and these discussions will vary greatly based on type of partnership, individual circumstances, environmental characteristics, and resource availability. We hope that our new data, in addition to adapting existing guiding tenets for GHP engagement [1, 13, 14] can help GHPs find sustainable solutions to virtual engagement during an unprecedented historical time.

Our study has several limitations. First, as an initial exploratory survey, we pursued convenience sampling, which meant it was not possible to document response rate to the survey. Second, we chose not to include participants from the EEA due to lengthy ethical review processes to meet European data protection requirements, an issue we plan to address in future surveys. Third, to focus on gathering baseline data and to not exclude respondents in LMIC who may not have had access to VGHPIs at the time of the survey, we did not include questions about specific virtual activity details or lessons learned from engagement; these topics, which we felt to be outside the scope of this exploratory survey, will be a focus of our group’s future research. Finally, partial survey response may have affected results, though this was only 10% of the respondents; the pattern was determined to be missingness completely at random and did not disrupt results. Despite the limitations, we believe we gained valuable insight into a variety of GHPs at a key moment during the COVID-19 pandemic. Our baseline data is important to guide future mixed-method and qualitative work about VGHPIs, to provide a “before” comparison to help other groups with similar goals evaluate the impact of the pandemic on their GHP activities, and to foster meaningful discussion within partnerships. Future study should likewise focus on the differences between the many varieties of GH partnerships in LMIC versus HIC setting.

## Conclusions

GHPs were significantly disrupted by the COVID-19 pandemic, in particular their funding, communication, resource access, and activities, but the ways in which these disruptions affected LMIC versus HIC partners were disparate. Our survey of GHPs in both HIC and LMIC identified a strong enthusiasm for VGHPIs despite several key barriers. VGHPIs may bridge existing gaps and maximize gains of GHPs for enhanced bidirectionality, transparency, and equity in GHPs. Future study is needed to measure and guide best practices in this rapidly developing landscape.

## Supplementary Information

**Additional file 1. Figure 3:** Perceived barriers and acceptability of virtual global health partnership initiatives (VGHPIs) among respondents.

## Data Availability

The datasets used and/or analyzed during the current study are available from the corresponding author on reasonable request.

## Abbreviations

EEA: European Economic Area
GH: Global health
GHP: Global health partnership
HIC: High-income country
LMIC: Low- to middle-income country
VGHPI: Virtual global health partnership initiative
