# Supplementary material for: Perceived roles, benefits and barriers of virtual global health partnership initiatives: a cross-sectional exploratory study
Source: Glob Health Res Policy. 2022 Apr 28;7:11. doi: 10.1186/s41256-022-00244-4 (PMC9046069; doi:10.1186/s41256-022-00244-4)
Supplement: Supplementary file 1 — Additional file 1. Figure 3: Perceived barriers and acceptability of virtual global health partnership initiatives (VGHPIs) among respondents. [file 41256_2022_244_MOESM1_ESM.docx]

**Figure 3: Perceived barriers and acceptability of virtual global health partnership initiatives (VGHPIs) among respondents**

Abbreviations: VGHPI = virtual global health partnership initiative

Abbreviations: VGHPI = virtual global health partnership initiative
